# Experimental Study of the Triplet Synchronization of Coupled Nonidentical Mechanical Metronomes

**DOI:** 10.1038/srep17008

**Published:** 2015-11-24

**Authors:** Ji Jia, Zhiwen Song, Weiqing Liu, Jürgen Kurths, Jinghua Xiao

**Affiliations:** 1 School of Science, Beijing University of Posts and Telecommunications, Beijing 100876, China; 2 State Key Lab of Information Photonics and Optical Communications, Beijing University of Posts and Telecommunications, Beijing 100876, China; 3 School of Science, Jiangxi University of Science and Technology, Ganzhou 341000, China; 4 Institute of Physics, Humboldt University Berlin, Berlin D-12489, Germany; 5 Potsdam Institute for Climate Impact Research, Telegraphenberg, Potsdam D-14415, Germany; 6 University of Aberdeen, Institute for Complex Systems and Mathematical Biology, Aberdeen, AB24 3UE, United Kingdom; 7 Nizhny Novgorod State University, Department of Control Theory, Nizhny Novgorod, 606950, Russia

## Abstract

Triplet synchrony is an interesting state when the phases and the frequencies of three coupled oscillators fulfill the conditions of a triplet locking, whereas every pair of systems remains asynchronous. Experimental observation of triplet synchrony is firstly realized in three coupled nonidentical mechanical metronomes. A more direct method based on the phase diagram is proposed to observe and determine triplet synchronization. Our results show that the stable triplet synchrony is observed in several intervals of the parameter space. Moreover, the experimental results are verified according to the theoretical model of the coupled metronomes. The outcomes are useful to understand the inner regimes of collective dynamics in coupled oscillators.

Coupled dynamical systems which exhibit rich collective behavior are widely explored in biological^1^,^2^, mechanical^3^ or electrical, synthetic genetic networks^4^. With increasing strength of interactions between units, the whole coupled system may transit from some incoherent state to coherent ones, i.e. synchronization^5^,^6^,^7^ and oscillation death^8^,^9^. Based on the specific form of the coherent motion, various types of synchronization, including complete synchronization^5^, phase synchronization^6^, partial phase synchronization^7^, etc, are revealed in coupled oscillators. Since synchronization is one of the inner regimes of collective dynamics and pattern formation^10^,^11^,^12^, it remains a topic of interest in numerous theoretical and experimental studies and finds various applications.

Among, phase synchronization, defined as the locking of phases between interacting oscillators with different natural frequencies, is strongly relevant to practical situation. Simply, two interacting oscillators are deemed to be *n*:*m* phase synchronization if the following condition is fulfilled for t is larger than a transient time T.




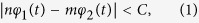




where *n* and *m* are some integers and *C* is a rather small constant. Analyzing complex synchronization patterns in multi-frequency systems have been deeply applied in various fields, especially widely in biological systems such as the interaction of respiratory, cardiac and brain activities^13^. When considering a large number of interacting oscillators with complex interacting network structure and random natural frequency distribution, there are rich dynamics and patterns in the processes that the coupled system transits from incoherent states to full phase locking states with increasing coupling interactions. Before reaching full phase locking state (any pair of coupling units satisfy the phase locking condition), the system may become a partial phase synchronization regime^7^, i.e. some pairs or groups of oscillators are phase locked while others are not which forms several synchronous clusters. To reveal all synchronous states of a network efficiently, Kralemann *et al.*^14^ defined a synchronous index to detect a triplet synchronization which is realized when *triplets* of interacting oscillators adjust their phases and frequencies so that the following conditions are fulfilled for t is larger than a transient time T,




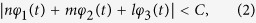




where the integers *n*, *m*, *l* can be both positive and negative and *C* is a small constant. However in parallel, the conditions of the pair-wise synchrony equation (1) may *not* be satisfied for any pair of units. Although, triplet synchronization is theoretically predicted and detected in oscillator networks from observed data, it is expected to reveal various pattern formations of coupled oscillators and to contribute to research in neuroscience based on the binding by-synchrony hypothesis^15^. To our best knowledge, no experimental observation on triplet synchronization has been observed so far. Experimental discussion on the triplet synchronization is important for various applications, such as the interaction of different brain regions, where oscillations with a hierarchy of frequencies are ubiquitous.

Coupled pendulums are deemed as a paradigmatic model of exploring the dynamics of coupled systems since the pioneering work of Huygens. Recently, many scientific teams carried out a variety of experimental research work, such as Wu Ye *et al.*^16^ showed a relationship theoretically and experimentally between the initial values and the friction damping force and the stable synchronous states of coupled metronomes system. Oliveira *et al.*^17^ experimentally explored Huygens synchronization in two clocks hanging from an aluminum rail fixed to a masonry wall. Hu *et al.*^18^ studied the synchronous behavior of three coupled metronomes, discovering a variety of synchronous states and the envelope synchronization phenomenon. Martens *et al.* found Chimera states in coupled metronome systems^19^, etc^20^,^21^. Therefore, coupled pendulums are a promising candidate to observe triplet synchronization experimentally. We set up here a coupled system with three globally coupled pendulums and apply the synchronous index defined in Eq. (2) to observe the triplet synchronization.

In this article, we try to experimentally observe the triplet synchrony with the aid of the synchronous index. A model based on our experimental setup is built and analyzed to verify our experimental results.

## Experimental setup

**Figure 1:**
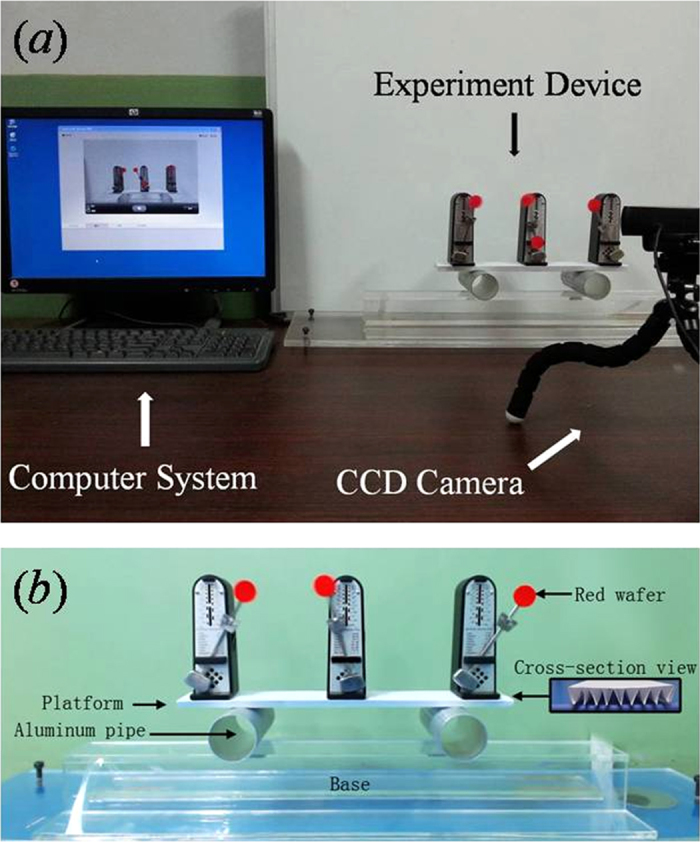
Experimental set up. (**a**) An overall view of the experimental system, including experiment device, CCD camera and computer system. (**b**) Front view of the experimental system recorded by the CCD camera, including the base, aluminum pipes, coupling board and metronomes. Several red wafers are pasted to mark the positions of desired parts.

Figure 1 shows the experiment platform which consists of three metronome units supported by a piece of folded A4 paper on two aluminum pipes, a CCD (Charge Coupled Device) acquisition system connected to a computer and software of LABVIEW. The metronomes in our experimental work are all the Taktell Piccolino (Series 890) manufactured by Wittner GmbH & Co.KG in Germany. In order to improve the accuracy and simplicity, the latest experimental system are ameliorated based on the previous system^18^,^19^,^20^,^21^,^22^,^23^. An organic glass base of hollow cuboids shape is used to ensure that the system will not produce deformation because of its own weight. Two aluminum pipes (with 39 mm inside diameter, 41 mm outside diameter, and 100 mm length) are put on and perpendicular to the base. The aluminum pipes have a lot of advantages, such as that their rolling friction and shape hardly change and they have lighter mass.

Since the total energy supplied by the metronome units is limited (last about 20 minutes), it is difficult to realize synchronization if the coupling strength is not sufficient large. In order to enhance the effect of coupling, the crux of the problem is to provide more energy or reduce unnecessary loss. Without changing the structure of metronome, a paper-made platform was applied to substitute the coupling board used in the previous works^16^,^18^. A few pieces of A4 paper are folded as undulating shape so that it is strong enough to support the metronomes. Thus, with the lightness of the coupling board, the energy of the system will not waste too much of the kinetic energy of the coupling board. As a result, the coupling strength is guaranteed strong enough to realize synchronization between metronomes on the coupling board.

Three metronomes are put on the coupling board and a red wafer is pasted at the end of each pendulum and on the coupling board to improve the accuracy of recognition for the CCD camera. Then the motion of the pendulums and the coupling board can be conveniently recorded by tracing the center of the red wafers. In order to get accurate data, a camera with a high frame rate is set up by which one can record videos with a resolution of 720p (1280*720 pixels) and a frame rate of 30 frames per second. The time series of the pendulum of each metronome are recorded by handling the videos.

In our experiments, the metronomes are numbered as 1 to 3 from left to right. By adjusting the equivalent lengths of pendulums, we may change the frequency of the metronome slightly. (Noted that there are some slight differences between the actual frequencies and the set values which are caused by the instrumental errors (about 0.4%) in the mechanical structure of the metronomes. Therefore, we use all measured values of frequencies other than nominal ones of the metronome).

To provide enough energy necessary for the coupling, we set a relatively high value of initial frequency of metronomes as *f*_1_=160 beats per minute (BPM) and *f*_1_=176 BPM, while adjusting the value of the initial frequency *f*_3_ of the 3^rd^ metronome from 120 BPM to 200 BPM and so 107 values of *f*_3_ are obtained. The time series for each initial frequency constellation are collected by the CCD camera, and the corresponding phases are calculated with the aid of a computer.

## Analysis Methods

The synchronous ratio and order parameter are both effective indicators verifying the synchronous behavior of system. In the work of Kralemann *et al.*^14^, the two indicators were combined and a special synchronous index was proposed as shown in Eq. (3) and (4). The pair-wise synchronization indices can be described as follows when two oscillators *i* and *j* are coupled:




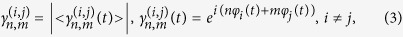




where 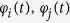 are the phases of the oscillators *i* and *j* respectively, *n* and *m* are integers, and <> is average on the time t. The oscillators *i* and *j* are considered as being *n*:*m* synchronized when the value of the pair-wise synchronization index 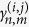 is equal to 1. Accordingly, when all three oscillators are coupled, the triplet synchronization indices can be calculated by equation (4):




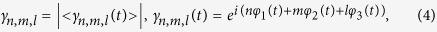




where 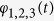 are the phases of the oscillators 1–3 respectively, *n, m* and *l* are integers and <> is average on the time t. Then the triplet synchronization indices 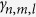 can be calculated for all possible integers of *n*, *m*, *l*. The state of the coupled system can be determined as the following cases. (1) If both the triplet synchronization index 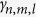 and all the pair-wise synchronization indices 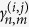 are approaching to 1, the coupled system is in complete phase synchronization other than triplet synchronization. (2) If the triplet synchronization index 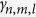 is approaching to 1, while the pairwise synchronization indices are small, the coupled system is in triplet synchronization. (3) If the triplet synchronization index 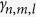 is equal to 0, at least two of the three phases are completely independent.

## Experimental Results

**Figure 2:**
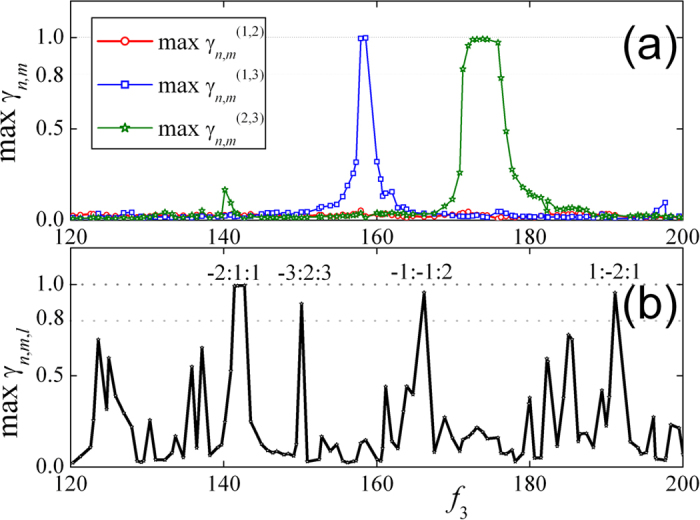
The distribution of synchronous indices in our experiments. (**a**) The distribution of the experimental pair-wise synchronous index in Eq. (3). The pair-wise synchronous indices between units 1, 2, 1, 3 and 2, 3, which are depicted as red, blue, and green line respectively, VS. the frequency *f*_*3*_ of the 3^rd^ metronome. (**b**) The triplet synchronous indices in Eq. (4) max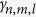 are shown as black line. The synchronous parameters *n*, *m*, *l* are labeled on the peaks of the corresponding frequencies.

According to the recorded data of the swing angle 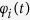 of the pendulums (the data is recorded after a transient time T = 300 seconds), we calculate all triplet and pair-wise synchronization indices for all 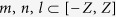 (with *Z *=* 5* in our experiment) and record the maximal value of the indices as 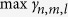 or 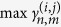. The maximal pair-wise synchronous indices between units (1, 2), (1, 3) and (2, 3) versus the frequency *f*_3_ of the 3^rd^ metronome are presented in Fig. 2(a), which are depicted as blue, red and green line respectively. For instance, when *f*_3_ = 158 BPM or *f*_3_ = 176 BPM, there are two intervals of 
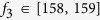
 BPM and 
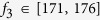
 BPM, where the pair-wise indices 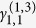 and 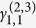 are approx to 1 respectively. Therefore, the coupled system is in pair-wise synchronous state between (1, 3) and (2, 3) when 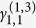 and 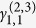 is larger than 0.8 as mentioned in ref. 14. In Fig. 2 (b), the maximal values of all triplet synchronous indices 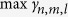 are shown as black line. It is obvious that there are four intervals of *f*_3_ where 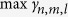 is approaching 1, while the pair-wise indices of the corresponding intervals of *f*_3_ are small (approaching zero). Therefore, the system reaches the triplet synchrony in those intervals of parameter *f*_3_. That is to say, any pair of metronomes is asynchronous but the whole system of the three metronomes is in a triplet synchronous state when the parameter *f*_3_ is in one of those four intervals. The corresponding integer parameters *n*, *m*, *l* are marked on the peaks of corresponding frequencies *f*_3_.

**Figure 3:**
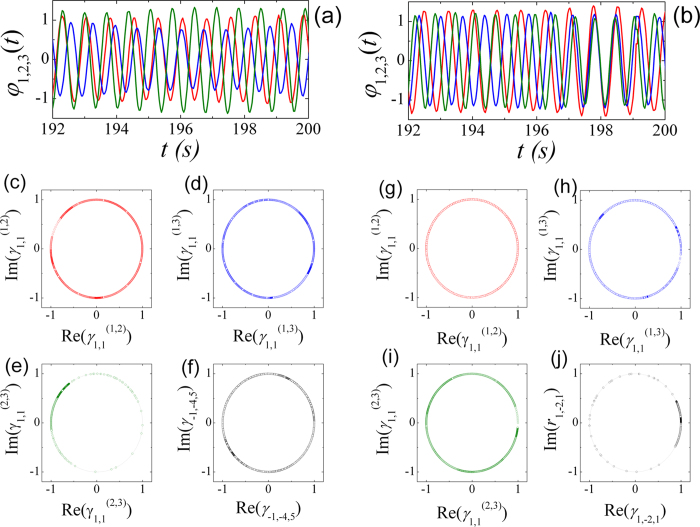
The time series of swing angle of three metronomes. The coupled system is in (**a**) pair-wise synchronization for 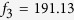 BPM and (**b**) triplet synchronous state for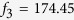 BPM, respectively. (**c**–**e**) The phase diagram of 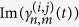 and 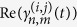 for (*i*, *j*) = (1, 2), (1, 3), (2, 3) respectively, (**f**) the phase diagram of 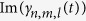 and 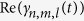 for a period of 8 seconds as shown in (**a**). (**g**–**i**) The phase diagram of 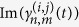 and 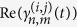, (**j**) the phase diagram of 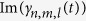 and 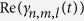 with *n*:*m*:*l* = 1:−2:1, for a period of 8 seconds as shown in (**b**).

Figure 3(a,b) present the time series of the swing angle 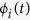 of the three metronomes when the coupled system is in a pair-wise synchronization and a triplet synchronous state respectively. It is difficult to figure out whether the pair-wise synchronization or the triplet synchronization is built between those three coupled metronomes only by the time series of the swing angles. However, if we calculate the values of 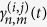 and 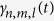 for a interval of time (8 seconds) respectively and dot them in the phase space of 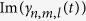 and 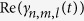 (or 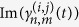 and 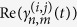), then the dots will distribute uniformly on a circle if no pair-wise synchronization (or triplet synchronization) is built, i.e., 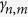 (or 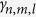) is small, otherwise, the dots are in a centralized distribution.

**Figure 4:**
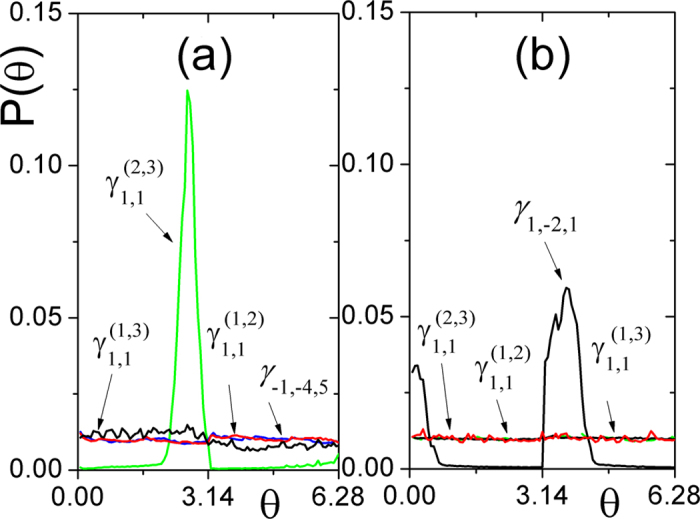
The phase distribution of the phase 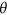 of (a) 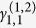 (red line), 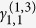 (black line), 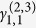 (green line),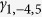 (blue line) corresponding toFig. 3(c–f), respectively. (b) 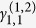 (red line), 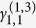 (green line), 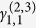 (blue line), 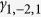 (black line) corresponding to Fig. 3(g–j).

With the aid of the phase space representation of 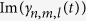 and 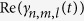 (or 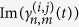 and 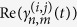), it is convenient to observe whether the coupled metronomes are in triplet or pair-wise synchronization. Figure 3(c–e) present the phase diagram of 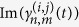 and 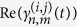 for (*i*, *j*) = (1, 2), (1, 3), (2, 3) respectively with 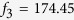 BPM in experiment. The dots of 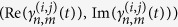 for (*i*, *j*) = (2, 3) distribute centrally on the circle, while those of (*i*, *j*) = (1, 3) and (1, 2) distribute uniformly on the circle. Therefore, pair-wise synchronization is only built between metronomes 2 and 3, which coincide well with the results presented in Fig. 2(a). However, the dots of 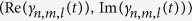 are also distributed uniformly on the circle. Hence, there is no triplet synchronization between the coupled three metronomes, where the values of *n*, *m*, *l* are determined when the value of 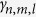 is maximal. Meanwhile, we present the experimental results for 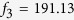 BPM in Fig. 3 (g–j) which are corresponding to that in Fig. 3 (c–f). Obviously, there is no pair-wise synchronization but a triplet synchronization with 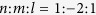. What should be mentioned is that there are small amount of scatter dots in Fig. 3(e,j) which is caused by the sampling error of the CCD. As a result, the amount of data shown on the circle is limited (here we present data of 8 seconds) to observe the collective dynamics clear. To exhibit the collective dynamics more efficiently and without being influenced by the scatter dots caused by the sampling errors, the phase angle 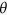 of the dot on the circle is defined as 
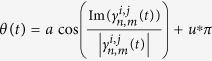
, with *u = 0* if 
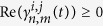
 and *u = 1* otherwise. The phase locking can be judged according to the curve of the possibility density 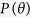 of corresponding 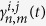. If the coupled system is in phase locking then the curve of corresponding 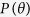 has a peak otherwise it will be uniformly distributed. Obviously, there is a peak of 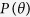 around 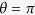 for 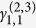 and a uniform distribution of 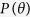 for 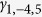, 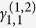, 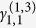 as shown in Fig. 4 (a) when the coupled system is in pair-wise synchronization as Fig. 3(a). However, there is uniform distribution of 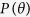 for 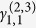, 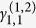, 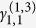, and peaks of 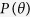 around 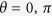 for 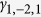 as shown in Fig. 4 (b) when the coupled system is in triplet synchronization as Fig. 3(b).

### Theoretical model and numerical results

**Figure 5:**
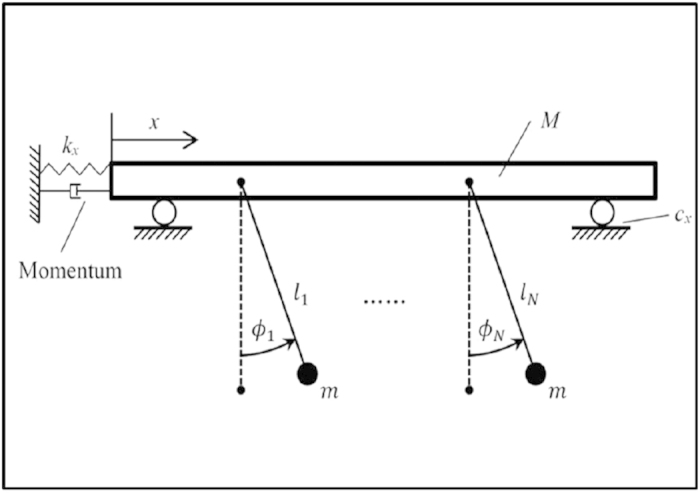
Theoretical model of coupled metronomes.

To reveal the observed triplet synchronization in the experimental setup, a theoretical model derived from the experimental devices is analyzed^24^,^25^. The experimental devices are abstracted from that in refs 26, 27, 28, 29 as shown in Fig. 5 where several pendulums with the same mass are coupled through a board, which can move horizontally. All pendulums are swinging around the fixed point above and in a common upright plane. The length and swinging angles of pendulums are denoted by *l*_*i*_ and 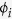. Two aluminum pipes are set parallel under the coupling board and thus the board can move horizontally. The displacement of the board is denoted by *x*; *c*_*x*_ and *k*_*x*_ are damping and linear force respectively.

Without the damping and driving force, the Lagrange equation of the system is as follows:




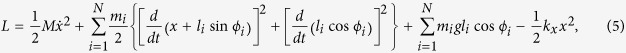




and can be simplified into,




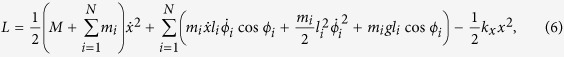




where *M* is the mass of the board, *m*_*i*_ = 1 is the mass of the pendulum, *x* is the displacement of the board with 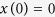. 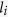 and 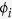 are the length and angle of the *i*th pendulum, *g* is the gravity. We define the right direction as the positive direction. In Huygens experiments, the coupling board was limited by magnetic substance, and therefore the parameter 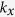 is remained to repeat the earlier work and it is set as 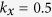.

With the effects of damping and driving force, which is caused by the escapement mechanism of the metronomes, the dynamic equation of the coupled system can be solved:




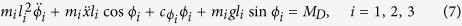







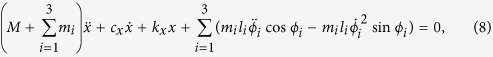




where 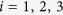, 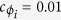 is the damping of each pendulum, and 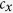 is the damping of the coupling board. 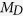 is the driving force. When the rod of the pendulum swings cross zero and the angle is less than 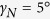, the driving force is produced as follows:




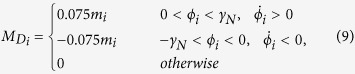




The fourth order Runge-Kutta method is applied and the interval time is set as 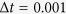 in our simulations. Exceptionally, setting 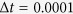 is helpful to solve the problems about Lyapunov exponent. At the time 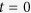, the initial velocities of all objects and displacement of the coupling board are set as zero, and the initial values of the swinging angles are set randomly. The coupled system will go into stable states usually after 300 seconds of transient time.

**Figure 6:**
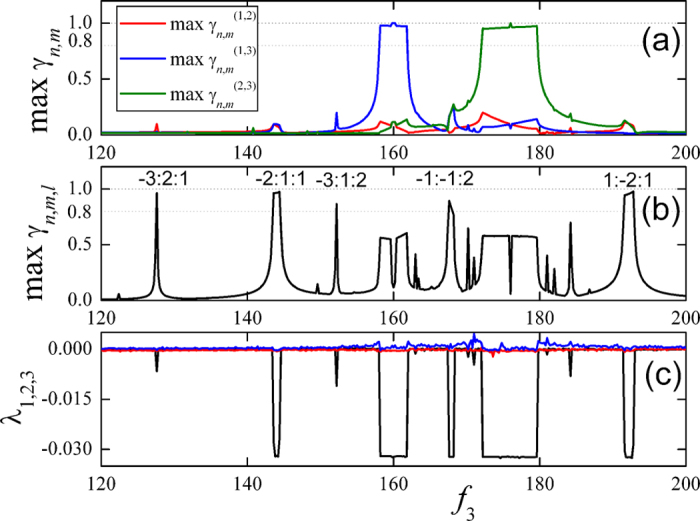
The distributions of synchronous indices in numerical simulation. (**a**) The distributions of pair-wise synchronous indices, and the max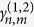, 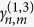 and 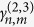 are plotted by red, blue and green curves respectively. There are two sections where the pairwise indices are greater, around the frequencies of 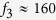 BPM and 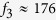 BPM, and it shows that the metronomes (1, 3) or (2, 3) of the system are pair-wise synchronous respectively and synchronous parameters are 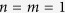. (**b**) The triplet synchronous indices, plotted by the black curve. There are 5 sections where 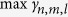 is approaching 1 (larger than 0.8), i.e. the system reaches triplet synchrony, and the synchronous ratios are labeled on the peak of the corresponding frequencies. (**c**) The distribution of the largest three of Lyapunov exponents. When the third largest Lyapunov exponent 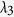 is negative, the system is phase-locked.

While simulating equations (7) and (8) numerically, we fix the parameters as follows, 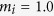, 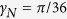, 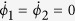, 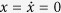, 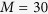 and 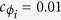. In order to compare them with our experimental results, we firstly fix the frequencies of two metronomes as *f*_1_=160 BPM and *f*_1_=176 BPM, and adjust *f*_3_ from 120 to 200 BPM gradually. The length of the pendulum can be determined according to the equation of 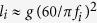. Then the pair-wise synchronous indices and the triplet synchronous indices can be calculated respectively according to the recorded time series of 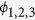. The total time of the recorded time series of phase are 500 seconds after a transient time *T = 300 *seconds. The results are not concerned with the length of the time interval. For example, the results of 2000 seconds and of 5000 seconds are the same as that of 500 seconds. For each value of *f*_3_, we determine the maximal synchronous index among all possible sets of n, m (or n, m, l) in a range of [−Z, Z] with Z = 5. If the value of 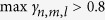, and the maximal value of 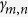 is small, then that triplet synchronization is built up between the coupled metronomes. Figure 6(a) shows the distributions of pair-wise synchronous indices, and the max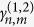, 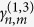 and 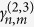 are plotted by red, blue and green curves respectively. There are two intervals of parameter *f*_*3*_ where the pair-wise indices are approaching to 1, around the frequencies of 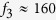 BPM and 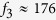 BPM. Therefore, the metronomes (1, 3) or (2, 3) of coupled system are in a pair-wise synchronous state respectively with the synchronous parameters 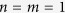. Accordingly, the maximal value of triplet synchronous indices are presented in Fig. 6(b) and plotted by black curve. There are 5 intervals of *f*_3_ where 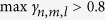, with the parameters n,m,l as marked on the corresponding intervals (such as −2:1:1 indicates that the synchronous parameters are 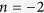, 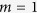, 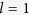 which realize the maximal value of the triplet index for given *f*_3_). However, the n:m pair-wise synchronous are excluded only in the range of [−Z, Z] with Z = 5 for the sake of consistent with the experimental one. It is necessary to exclude higher order of n:m pair-wise synchronous. Therefore, we calculate and record the maxima of the pairwise synchronous index for all possible n:m in the interval of [−100, 100] with parameter *f*_*3*_ being in stage when the the maximal value of the triplet indices are above 0.8. The maximal pairwise synchronous index is 
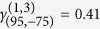
 which is less than 0.8. Even larger range of n, m, for example, [−300, 300], the maximal pairwise synchronous indices are not larger than those when m and n are in the range of [−100, 100]. Therefore, we may deduced that triplet synchronization is stable for given range of m and n. However, it is a time-consuming work for even larger range of n, m.

The largest three Lyapunov exponents are an effectively indicator of phase synchronization between the coupled system. Since the metronome is a mechanical system which is driven by discontinuous force from a spring, the standard algorithm does not work for the discontinuous dynamical equation. Thus the largest three of them, 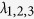, are calculated based on the algorithm introduced in refs 30,31. To exclude the effects of statistical fluctuation, 20000 seconds of time series are recorded to calculate the Lyapunov exponents. The Lyapunov exponents are stable when the time increases from 10000 seconds to 20000 seconds. In Fig. 6 (c), the largest three Lyapunov exponents are presented for different values of 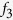. Phase synchronization is characterized by a negative value of the third largest Lyapunov exponent *λ*_3_. In the intervals of triplet or pair-wise synchrony, two of the largest three exponents are zero and the other one is negative and it shows that the system is quasi-periodic at these moments. In other frequencies, none of the three exponents are negative and at least two of them are approaching to zero, so the system is in high dimensional quasi-periodic or chaotic state.

**Figure 7:**
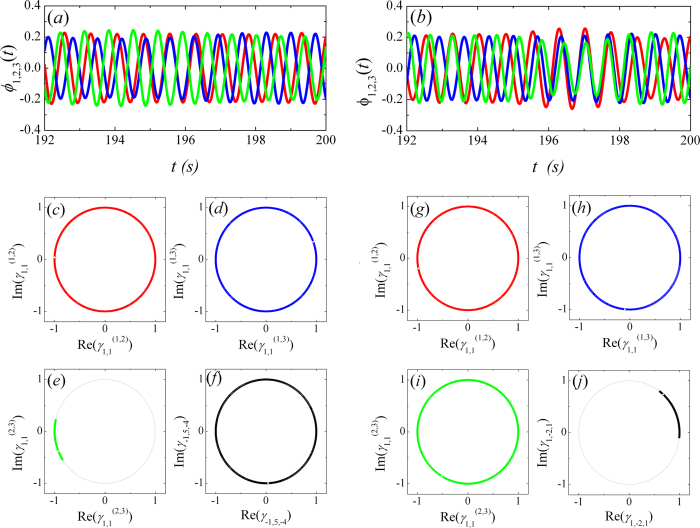
Numerical results of the time series of metronomes’ swing angles. The coupled system is in (**a**) pair-wise synchronization for 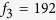 BPM and (**b**) triplet synchronous state for 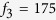 BPM, respectively. (**c**–**e**) The phase diagram of 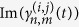 and 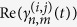 for (*i*, *j*) = (1, 2), (1, 3), (2, 3) respectively, (**f**) the phase diagram of 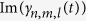 and 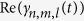 with *n:m:l* *=* *−1:5:−4* for a period of 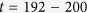 s as shown in (**a**). (**g–i**) The phase diagram of 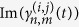 and 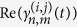, (**j**) the phase diagram of 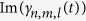 and 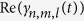 with *n*:*m*:*l* = 1:−2:1, for a period of 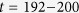 s as shown in (**b**).

To compare the numerical results with the experimental ones, we recorded the time series of the coupled metronomes’ angle for *f*_3_ = 192 BPM and *f*_3_ = 1752 BPM, respectively. We also cannot determine whether the coupled system is in triplet or pair-wise synchrony only from the time series. However, the phase space diagram of 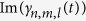 and 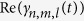 (or 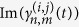 and 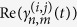) present clear relationships between the coupled metronomes. Obviously, when the value *f*_3_ = 192 BPM, pair-wise synchronization is built between metronomes 2 and 3, since the dots are collected in a small range on the circle as shown in Fig. 7(e). However, there is no triplet synchronization between the three coupled metronomes, since the dots are distribute uniformly on the circle as shown in Fig. 7(f) when *n, m, l* has value (−1:5:−4) when 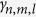 is maximal. When the value 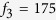 BPM, there is no pair-wise synchronization between the metronomes 1,2, 1,3, and 2,3, since the dots distribute uniformly on the circle as shown in Fig. 7(g–i). However, triplet synchronization with *n:m:l = 1:−2:1* is built between them as shown in Fig. 7(j).

**Figure 8:**
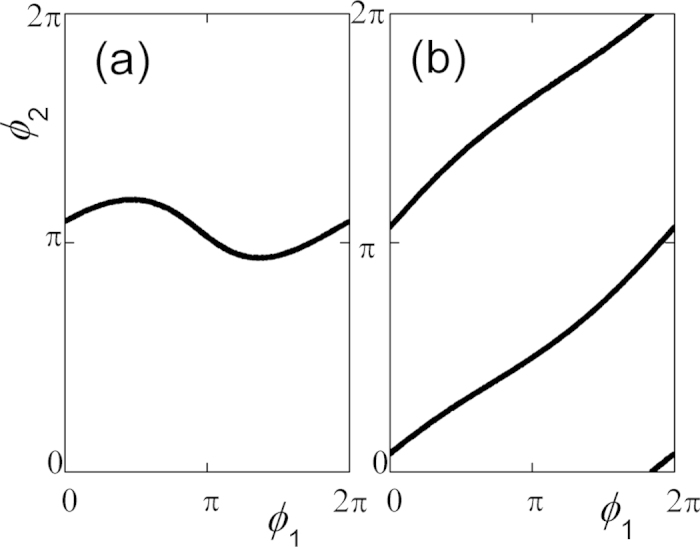
A Poincare map of system Eq. (7) (8). ** (a)** For fixing 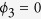 and *f*_3_=175 BPM, 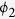 keeps constantly with small fluctuation as 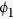 increases. There is pairwise synchronization between metronomes 2 and 3 while without pairwise synchronization between metronomes 1 and 2. (**b**) For fixing 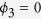 and *f*_3_=192 BPM, both 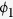 and 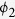 keep increasing from zero to 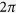 simultaneously, remaining, however, in a functional relationship 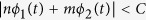 with *n:m = 1:−2*, this is an example of a triplet-synchronous state.

Another approach to verify the motion states is via Poincare maps which are got by recording the values of *ϕ*_1_ and *ϕ*_2_ when *ϕ*_3_ = 0 as shown in Fig. 8. The Poincare map (in Fig. 8(a), 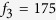 BPM) is a horizontal line with small variation in the parameter space of *ϕ*_1_ versus *ϕ*_2_ when *ϕ*_3_ = 0. Hence, it is confirmed this way that the coupled system built pair-wise synchronization only between metronomes 2 and 3 while there is no pair-wise synchronous between *ϕ*_1_ and *ϕ*_2_. As a result, *ϕ*_1_ influences the pair-wise synchronization between *ϕ*_2_ and *ϕ*_3_ which leads to a small modulation or perturbation. However, the Poincare map (in Fig. 8(b) 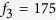 BPM) shows that both phases *ϕ*_1_ and *ϕ*_2_ vary from zero to 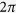 and remain in a functional relationship 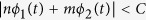 with 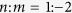 when *ϕ*_3_ = 0. Hence, there is no pair-wise synchronization between *ϕ*_2_ and *ϕ*_3_, *ϕ*_1_ and *ϕ*_3_ according to the fact that *ϕ*_1_ and *ϕ*_2_ vary from zero to 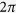 when *ϕ*_3_ = 0. If the coupled metronome system is triplet synchronous with *n:m:l = 1:−2:1*, then 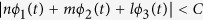. If *ϕ*_3_ = 0, it comes to the result of 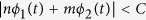 with 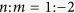 as shown the Poincare map in Fig. 8(b).

Comparing Figs 2 and 6, it is obvious that the numerical analyses are consistent with our experimental results and the theoretical model is effective to describe the experimental process. However, it should be mentioned that there are some differences between them. Firstly, there are 5 intervals of triplet synchrony, in numerical results but only 4 of them are observed in our experiments. By checking our experimental results carefully, we find that there is a smaller peak at the frequency of 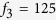 BPM, and its maximum value is less than 0.8. Next, the sections of pair-wise synchrony, which are observed in our experiments, are smaller than the simulated ones, especially at the frequency of 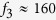 BPM. A reasonable explanation is that the interval of adjacent frequencies cannot be adjusted as small enough as the width of the peak in the experiments, thus the exact values of *f*_3_ corresponding to the peak of the index cannot be observed. This can be explained as that the results are almost the same when *f*_3_ is larger, but the synchronous sections are much more narrow in the experiments when *f*_3_ is smaller. A possible reason is, due to the escapement mechanism of the metronomes, that the lower frequencies cause less energy in unit time and the coupling effect is decreased. To verify the reason of the difference as described above and explore the universal rule of this behavior, the distributions of more extensive and various parameter spaces are discussed.

**Figure 9:**
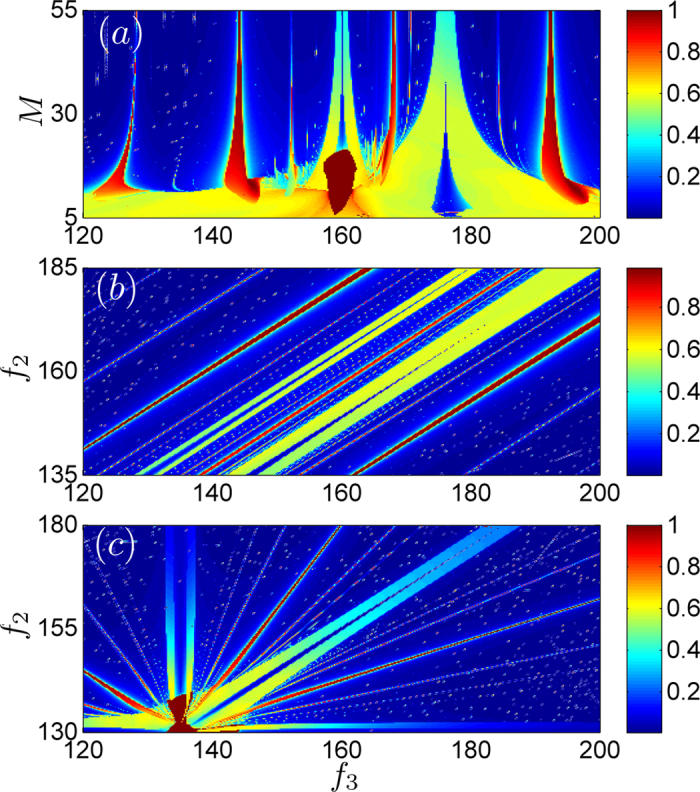
The parameter spaces of the synchronous indices Eq. (4) of system Eq. (8). The different colors denote the value of indices. (**a**) The effect of the mass of coupling board vs *f*_*3*_, fixing *f*_1_=160 BPM and *f*_1_=176 BPM. (**b**) The effect of frequencies of the first two metronomes and the third one, by fixing 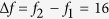 BPM. (**c**) The effect of the frequencies of the 2nd and 3rd metronomes, by fixing *f*_1_=160 BPM.

Since the mass of the coupling board affects the strength of the coupling, it is necessary to investigate the effect of the mass of the coupling board on the synchronous index. As shown in Fig. 9(a), the ordinate denotes *M*, the mass of the board, and the abscissa denote *f*_3_, the frequency of the 3rd metronome. We find that the system shows extremely complex phenomena when the mass is small. With the increasing of the mass, only a few sections are maintained. When the mass is about 30, system will work as our theoretical analysis predicts. The frequency difference 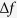 is fixed as 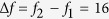 BPM, as shown in Fig. 9(b) (the ordinate denotes *f*_2_), and third one will influence the synchronous index. In addition, if the frequency of the 1st metronome is fixed as *f*_1_ = 160 BPM, we observe a relationship between *f*_*2*_ and *f*_*3*_ in Fig. 9(c).

Therefore, the distribution of the synchronous index is related to the frequencies of all metronomes. When the frequency differences are large enough, the behavior of the system depends on the coupling strength. We set the parameters in order to make the phenomena more clear and intuitive.

**Figure 10:**
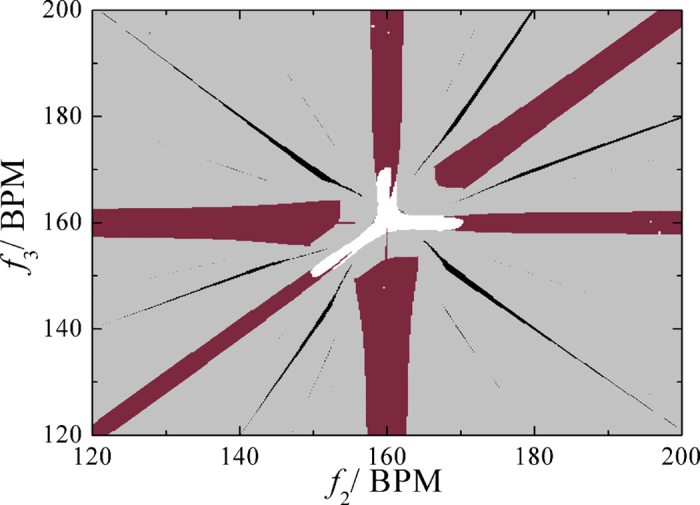
The distribution of synchronous index of system in the frequency space. When *f*_1_=160 BPM, white areas denote the complete synchronization of system, black ones denote the triplet synchronization. Wine ones denote the pair-wise synchrony and light gray ones are unlocked states.

Let us consider the synchronization indices based on the parameter spaces of *f*_2_ and *f*_3_ by fixing the value of *f*_1_ = 160 BPM. The results indicate that there are rich dynamics as the complete synchronization (white area), the triplet synchrony (black area), the pairwise synchrony (wine area) and unlocked states (light gray area) as shown in Fig. 10. Obviously, the probability for triplet synchrony is the smallest in the parameter spaces of *f*_2_ and *f*_3_.

To sum up, it is obviously that a very tiny difference in the value of parameters in the model system may lead to a deviation from the synchronization states. In consequence, taking inevitable instrumental error into account, the numerical analyses can be considered to be consistent with our experimental results as well as in the theoretical model. Based on the method of synchronous index, the triplet synchrony is observed in the experimental system of coupled metronomes.

## Discussion

In this paper, according to the theory of synchronization, an oscillation system of coupled metronomes was set up and triplet synchrony is discovered in a real experiment. By establishing the theoretical model and simulating the system numerically, we obtain consistent findings in experiments as well as theoretical models. By expanding the parameter spaces, we uncover more abundant nonlinear dynamic behaviors of coupled metronomes. It has been validated that the method of synchronous index is a useful tool to study experimental systems. Moreover, we propose a more direct method to determine triplet synchronization and pair-wise synchronization by a phase space representation of 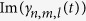 and 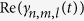 (or 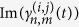 and 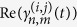). This approach can be used as a basis for applications and to detect such synchronization in various fields such as engineering, neuroscience, biology, etc. The discovery of triplet synchrony in our experimental system will help us to explore the physical mechanism of complex synchronization patterns in other real systems as well. It is hoped to play a role of guidance and construction in complicated synchronization behaviors in future.

## Methods

### Experiments

For the convenience of CCD acquisition, the bobs of the pendulums are pasted with red wafers respectively. The model of the metronome is Series 890 by DE Taktell with a mass (94 g). Its energy is supplied by a hand wound spring. The frequency of the metronome can be adjusted by changing the position of the mass on the pendulum bob, which denotes that the equivalent pendulum length is changed. The standard settings of the metronome’s frequency range from 40 ticks per minute (largo) to 208 ticks per minute (prestissimo), but not limited to the scale. The supporting folded A4 paper is light (4.366 g), since the pendulum bob’s swing direction is perpendicular to the aluminum pipes axes, a bidirectional coupling between the metronomes is generated via the folded paper, which has a tuning impact on the metronomes. Two parallel aluminum pipes (with inner (external) diameter 39 mm (41 mm)) support the folded A4 paper. Under the aluminum pipes there is a horizontal adjustment equipment.

### Simulations

The equations (1) and (2) are solved by the 4th-order Runge-Kutta method with a time step of 0.0001. The following parameters remain unchanged in the next parts of numerical calculation. We fixed the parameters 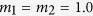, 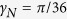, 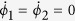, 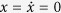, 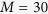 (calculated according to the experimental data) and 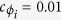.

## Additional Information

**How to cite this article**: Jia, J. *et al.* Experimental Study of the Triplet Synchronization of Coupled Nonidentical Mechanical Metronomes. *Sci. Rep.* **5**, 17008; doi: 10.1038/srep17008 (2015).
